# Claudin-4 Deficiency Results in Urothelial Hyperplasia and Lethal Hydronephrosis

**DOI:** 10.1371/journal.pone.0052272

**Published:** 2012-12-21

**Authors:** Harumi Fujita, Yoko Hamazaki, Yumi Noda, Masanobu Oshima, Nagahiro Minato

**Affiliations:** 1 Department of Immunology and Cell Biology, Graduate School of Biostudies, Kyoto University, Kyoto, Japan; 2 Graduate School of Medicine, Kyoto University, Kyoto, Japan; 3 Department of Nephrology, Tokyo Medical and Dental University, Tokyo, Japan; 4 Division of Genetics, Cancer Research Institute, Kanazawa University, Kanazawa, Japan; The University of Manchester, United Kingdom

## Abstract

Claudin (Cld)-4 is one of the dominant Clds expressed in the kidney and urinary tract, including selective segments of renal nephrons and the entire urothelium from the pelvis to the bladder. We generated *Cldn4* ^−/−^ mice and found that these mice had increased mortality due to hydronephrosis of relatively late onset. While the renal nephrons of *Cldn4* ^−/−^ mice showed a concomitant diminution of Cld8 expression at tight junction (TJ), accumulation of Cld3 at TJ was markedly enhanced in compensation and the overall TJ structure was unaffected. Nonetheless, *Cldn4* ^−/−^ mice showed slightly yet significantly increased fractional excretion of Ca^2+^ and Cl^−^, suggesting a role of Cld4 in the specific reabsorption of these ions via a paracellular route. Although the urine volume tended to be increased concordantly, *Cldn4* ^−/−^ mice were capable of concentrating urine normally on dehydration, with no evidence of diabetes insipidus. In the urothelium, the formation of TJs and uroplaques as well as the gross barrier function were also unaffected. However, intravenous pyelography analysis indicated retarded urine flow prior to hydronephrosis. Histological examination revealed diffuse hyperplasia and a thickening of pelvic and ureteral urothelial layers with markedly increased BrdU uptake *in vivo*. These results suggest that progressive hydronephrosis in *Cldn4* ^−/−^ mice arises from urinary tract obstruction due to urothelial hyperplasia, and that Cld4 plays an important role in maintaining the homeostatic integrity of normal urothelium.

## Introduction

Claudins (Clds), integral membrane proteins with 4 transmembrane domains, are crucial components of tight junctions (TJs), which act as a primary barrier to solutes and water between the apical and basal sides of epithelial cellular sheets [1], [2]. The claudin gene family consists of at least 24 members in mice and humans [2], [3]. Typically, multiple Clds are expressed in most types of epithelial cells, and the combination and ratio of different types of Clds in TJ strands may determine the permeability of each epithelial cellular sheet [3], [4]. The physiological importance of the barrier function of Cld-based TJs *in vivo* has been elucidated with the use of mice targeted for various Cld genes (*Cldns*), such as *Cldn1* in epidermis [5], *Cldn5* in blood–brain barrier [6], *Cldn11* in myelin and Sertoli cells [7], *Cldn14* in inner ears [8], [9], and *Cldn18* in stomach [10]. In addition, accumulating evidence reveals that Clds may function beyond being a simple barrier. In renal nephrons, for instance, Clds may regulate the reabsorption of selected cations or anions via a paracellular route between the tubular epithelial cells [11], [12], [13]. Among the Cld family members, Cld4 has been shown to act as selective paracellular channels for Cl^–^ [14] and barriers against cations [15] as well as to play a role with MUPP1 in the maintenance of a tight epithelium under hypertonic stress [16], [17] at least *in vitro*. Clds may also be directly or indirectly involved in the regulation of cell proliferation. It was demonstrated that *Cldn15* ^−/−^ mice developed mega-intestine due to the diffuse, nonneoplastic increase of upper intestinal epithelial cells [18]. Deregulated expression of Clds has been reported in many types of human tumors and may play a role in tumorigenesis as well [19]. For instance, Cld2 expressed in certain colon cancer cells played a significant role in their epidermal growth factor receptor (EGFR)-mediated cell proliferation, inasmuch as the knockdown of endogenous *Cldn2* compromised proliferation *in vitro* and tumorigenesis *in vivo* [20], [21]. More recently, we reported that Cld4 expressed on normal thymocytes was capable of enhancing T-cell receptor (TCR)-mediated ERK activation and proliferation in the fetal thymus organ culture [22].

Uroepithelium (urothelium) is a unique stratified epithelium that lines the urinary tract, including the renal pelvis, ureters, and bladder, and forms a highly distensible barrier that prevents unregulated substance exchanges between the urine and the blood supply. This process mainly involves uniquely differentiated umbrella cells [23]. While the urothelial cells show slow turnover rates (∼3–6 mo) at a steady state, they have enormous regenerative capacity and are rapidly restored following damage [23]. Urothelial plaques (uroplaques [UPs]) consisting of uroplakin particles play an important role in the barrier function of urothelium [24]. The urothelial cells express many types of Clds, including Cld4, throughout the layers in both humans and mice [23]; however, their exact function in the urothelium remains elusive. In current study, we demonstrate that *Cldn4* ^−/−^ mice develop progressive hydronephrosis as they age, resulting in increased mortality. Prior to the development of overt hydronephrosis, *Cldn4* ^−/−^ mice showed diffuse hyperplasia and thickening of the urothelium leading to urinary tract obstruction as revealed by intravenous pyelography (IVP), while the structure of TJs and the gross barrier effect were largely retained. Our results indicate that Clds play an important physiological role in maintaining the homeostatic integrity of the urothelium.

## Results

### Development of Hydronephrosis in *Cldn4^−/−^* Mice

**Figure 1:**
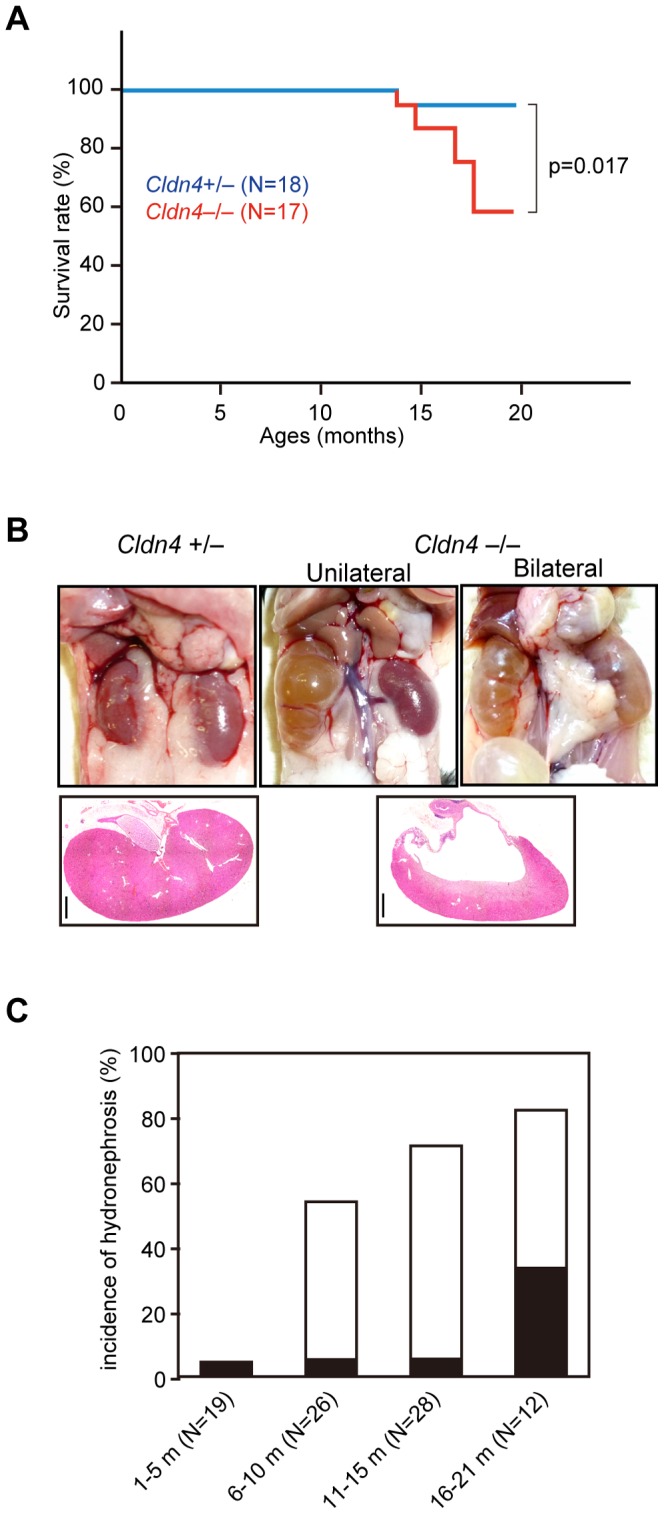
Development of hydronephrosis in *Cldn4* ^−/−^ mice. (*A*) Kaplan-Meier’s survival curves of *Cldn4^+/−^* (*N* = 18) and *Cldn4^−/−^* (*N* = 17) mice. (*B*) Macroscopic and microscopic appearances of kidneys in *Cldn4^+/−^* and *Cldn4^−/−^* mice. Two representative cases of unilateral and bilateral hydronephrosis in *Cldn4^−/−^* mice (8 months old) are shown. Bars; 1 mm. (*C*) Incidence of hydronephrosis at various ages of *Cldn4^−/−^* mice. Indicated numbers of *Cldn4^−/−^* mice were sacrificed at various ages, and the incidence of macroscopic hydronephrosis was examined. Open columns, unilateral hydronephrosis; closed columns, bilateral hydronephrosis. None of the *Cldn4* ^+/−^ mice developed hydronephrosis.

We generated loxP-floxed *Cldn4* targeted mice and mated them with *CAG-Cre* mice to develop germ-line transmittable *Cldn4*-deleted (*Cldn4* ^−/−^) mice (Figure S1). The *Cldn4* ^−/−^ mice were backcrossed with C57BL/6 (B6) mice for more than six generations. They were born in the expected Mendelian ratio and developed apparently normally. However, after the first year of age, *Cldn4* ^−/−^mice showed significantly increased mortality; the survival rate of *Cldn4* ^−/−^ mice at 20 months of age was 59% (10/17), whereas that of *Cldn4* ^+/−^ littermates was 94% (17/18) (Figure 1A). *Cldn4* ^−/−^ mice over 1 year of age frequently showed striking hydronephrosis with markedly dilated pelvis and severely compressed renal parenchyma (Figure 1B). Random sampling autopsy indicated that the macroscopic hydronephrosis was already evident in 54% of *Cldn4* ^−/−^ mice before 10 months of age and in 83% after 16 months (Fig. 1 *C*). While most of the hydronephrosis was observed unilaterally before 15 months, the proportion of bilateral hydronephrosis was markedly increased after 16 months *Cldn4* ^−/−^ mice, leading to increased mortality (Figure 1C). None of the *Cldn4* ^+/−^ littermates developed hydronephrosis until 21 months of age. Unaffected kidneys of *Cldn4* ^−/−^ mice were grossly normal without any signs of developmental anomaly upon histological examination, as were other organs.

### Compensatory Cld Expression and Preserved TJs and Barrier Function in *Cldn4^−/−^* Mice

**Figure 2:**
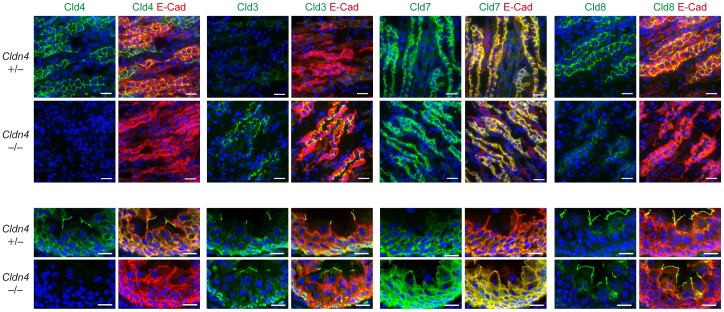
Compensatory accumulation of other Clds at TJs in the nephrons and urothelium of *Cldn4* ^−/−^ mice. Renal medullary regions (upper) and ureters (lower) of *Cldn4^+/−^* mice and *Cldn4^−/−^* mice (2 months old) with no evidence of hydronephrosis were three-color immunostained with anti-Cld4, anti-Cld3, anti-Cld7, or anti-Cld8 (green), along with anti-E-cadherin (E-cad) (red) and DAPI (blue). Bars, 50 µm (upper) and 20 µm (lower). Note the increased signal of Cld3 with diminished Cld8 signal at the cell-cell adhesion sites in renal medullary regions and increased Cld7 signal in ureter of *Cldn4^−/−^* mice.

**Figure 3:**
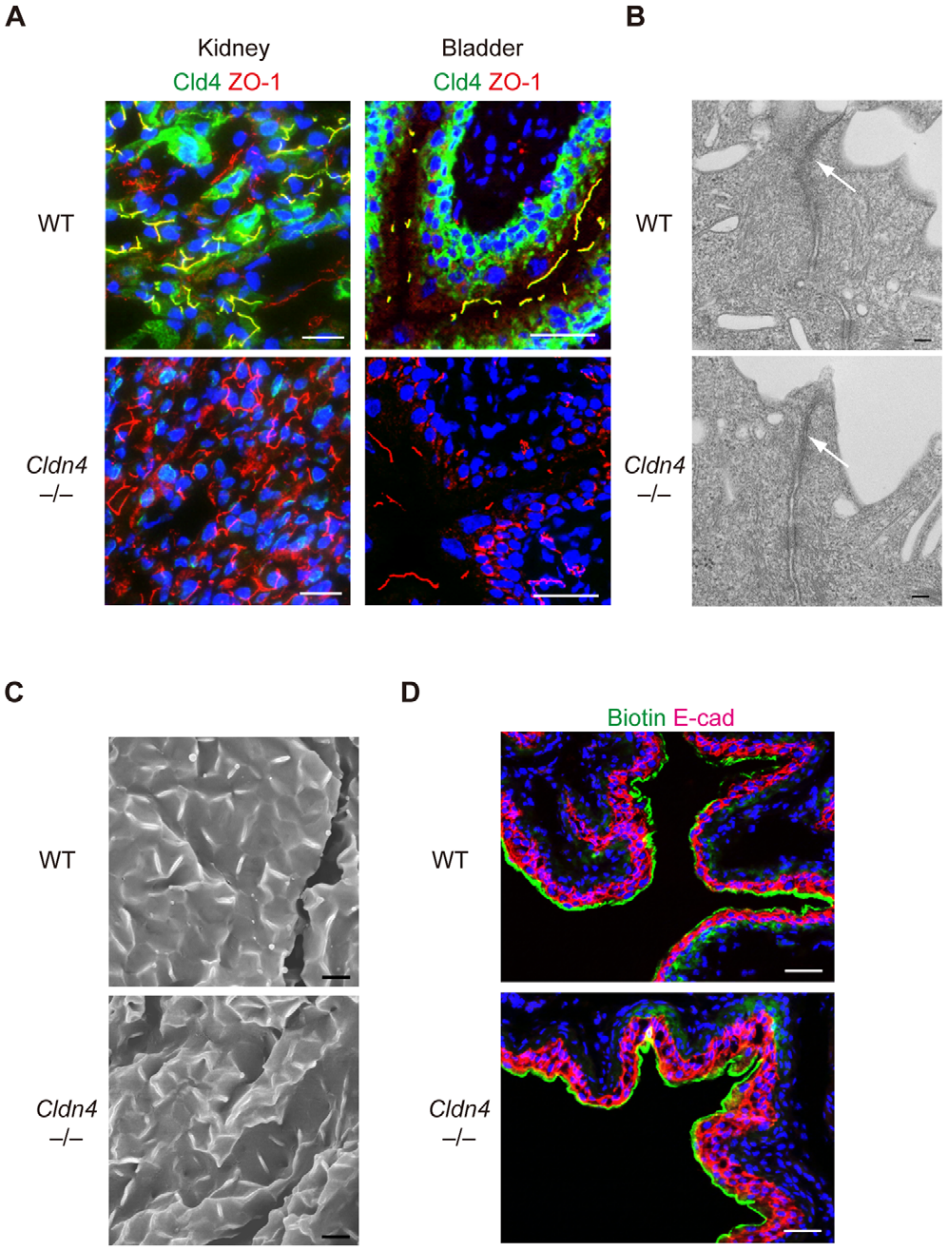
Preserved TJs and barrier function of TJs of *Cldn4* ^−/−^ mice. (*A*) Kidneys and bladders of WT and *Cldn4^−/−^* mice were immunostained with anti-Cld4 (green), anti-ZO-1 (red), and DAPI (blue). Bars, 50 µm. (*B*) Urothelium of ureters of WT and *Cldn4^−/−^* mice were subjected to ultrathin transmission electron microscopy. Bars, 100 nm. Arrows indicate TJs. (*C*) Urothelium of bladders of WT and *Cldn4^−/−^* mice were subjected to scanning electron microscopy. Bars, 5 µm. (*D*) WT and *Cldn4^−/−^* mice under anesthesia were injected with sulfo-NHS-SS-biotin into bladders, and 20 minutes later the bladders were dissected and 3-color immunostained with streptavidin (green) and E-cadherin (E-cad)(red) and DAPI (blue). Bars, 50 µm.

In the renal nephrons, Cld4 expression was detected selectively in the medullary AQP1^+^ cells corresponding to the thin descending loop of Henle [25], CIC-K^+^ thin ascending loop of Henle [26], TRPV5^+^ late distal convoluted tubule and connecting tubule [27], and AQP2^+^ collecting duct [28], sparing the cortical AQP1^+^ proximal tubules [25] and THP^+^ thick ascending loop of Henle [29] (Figure S2). Interestingly, although no Cld4 expression was detected in nephrons of *Cldn4^−/−^* mice as expected by immunostaining analysis, the collecting duct cells showed markedly enhanced signals of Cld3 and reduced Cld8 at TJs compared with those of *Cldn4* ^+/−^mice (Figure 2 *upper*). It was noted that Cld3 was apparently concentrated at TJs of collecting duct epithelial cells corresponding to Cld4 in *Cldn4* ^+/−^ mice (Figure 2 *upper*). Immunoblotting and quantitative RT-PCR analysis, however, revealed that the expression of Cld3 and Cld8 was unchanged at both protein and transcripts levels in kidneys (Figure S3A, B), strongly suggesting that the accumulation of Cld3 at TJs and reduced Cld8 signals of *Cldn4^−/−^* renal tubular epithelial cells was attributable to the molecular redistribution. In agreement with compensational localization of Cld3 at TJs in *Cldn4^−/−^*medullary nephrons, these tubular epithelial cells showed a characteristic expression profile of ZO-1 associated with TJs indistinguishable from WT cells (Figure 3A), suggesting that the TJ formation per se was hardly affected. In the normal urothelium, Cld4 was concentrated at TJs of the most apical umbrella cells and as well as being diffusely distributed throughout plasma membranes of underlying cell layers (Figure 2 *lower*). On the other hand, Cld8 expression was highly restricted at the TJs of umbrella cells, whereas Cld7 was detected mostly in the cells from intermediate to basal layers along the entire plasma membrane (Figure 2 *lower*). The urothelium of *Cldn4^−/−^* mice showed markedly enhanced signals of Cld7 at the TJs of umbrella cells, although the expression patterns of Cld3 and Cld8 were unchanged (Figure 2 *lower*). Again, expression of Cld7 as well as other Clds expressed in urothelium [30] were not altered at the total protein level (Figure S4). Accordingly, the TJ formation was unaffected in the *Cldn4^−/−^* urothelium as judged by both the ZO-1 expression profile and ultrastructural analysis (Figure 3A, B); UP formation on the surface of the bladder was also normal (Figure 3C). Furthermore, the bladders of *Cldn4^−/−^* mice showed a barrier effect for small molecular weight tracers comparable to that of WT littermates (Figure 3D). Altogether, these results suggest that the formation of TJs and the barrier function are largely conserved in the nephrons and urothelium of *Cldn4^−/−^* mice, most likely due to the compensatory relocalization of other specific Cld members at TJs.

### Impaired Renal Reabsorption of Selective Ions in *Cldn4^−/−^* Mice

**Figure 4:**
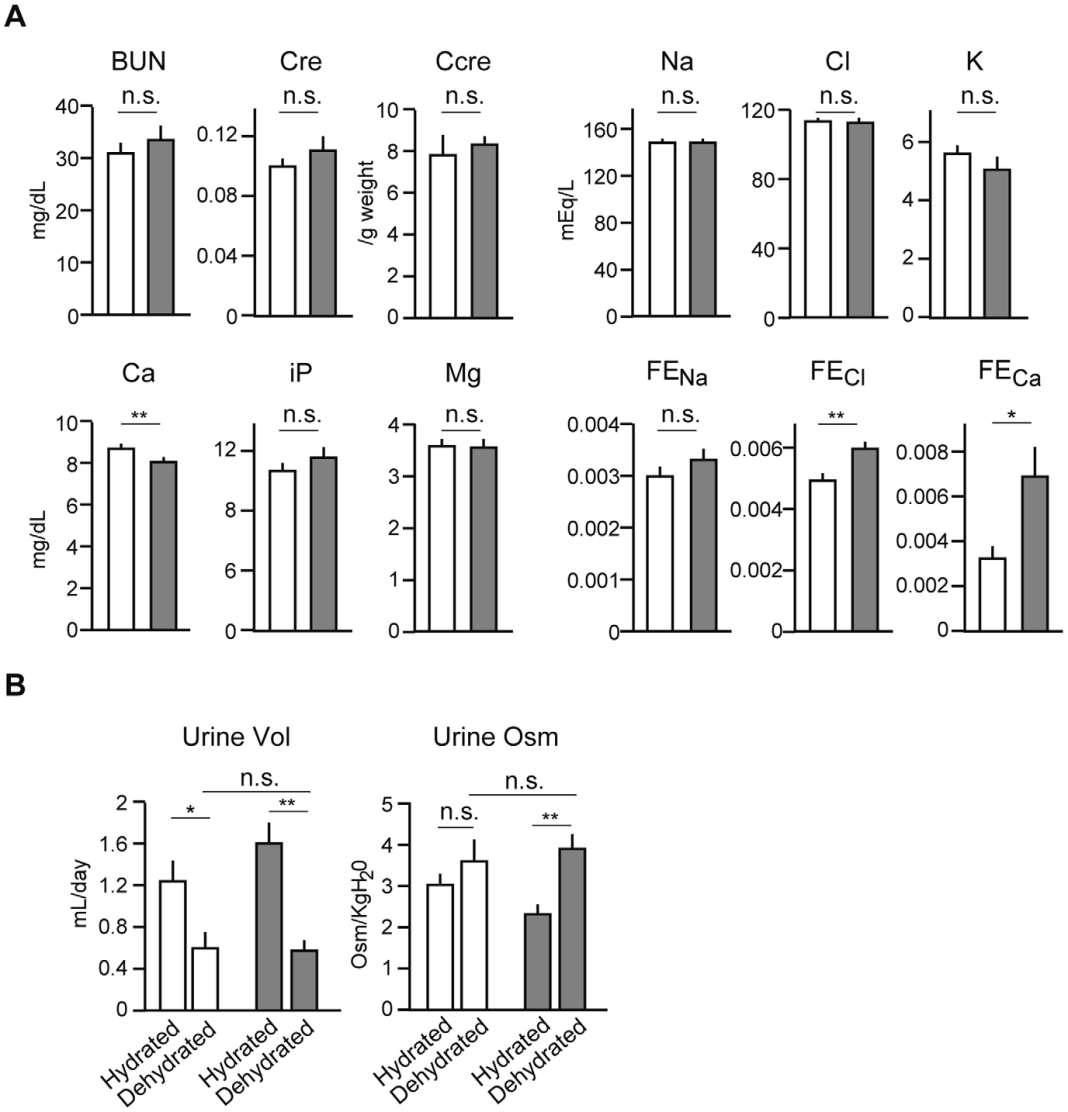
Impaired reabsorption of Ca^2+^ and Cl^−^ in *Cldn4^−/−^* mice. (*A*) Indicated ion concentrations in the urine and serum of WT (open columns) and *Cldn4^−/−^* (gray columns) mice (3 months old) with no evidence of hydronephrosis were analyzed, and the serum ion concentrations, creatinine (Cre), blood urea nitrogen (BUN), creatinine clearance (C_cre_), and ion fractional excretions (FE) were determined. The means and SE of five mice are indicated. n.s., not significant; *p<0.05; **p<0.01. (*B*) Total 24-hour urine volume and osmolality of WT (open columns) and *Cldn4^−/−^* (gray columns) mice before and after the water depletion for 24 hours were determined. The means and SE of five mice. n.s., not significant; *p<0.05; **p<0.01.

Prior to overt hydronephrosis, 3-month-old *Cldn4^−/−^* mice showed serum blood urea nitrogen and creatinine within normal ranges (Figure 4A). Also, the concentration of serum ions, including Na^+^, K^+^, Cl^−^, iP, and Mg^2+^, was unaffected except for a mild, yet significant decrease of Ca^2+^ (Figure 4A). The decrease of serum Ca^2+^ was paralleled by an increased Ca^2+^ fractional excretion (FE_Ca_), suggesting impaired reabsorption. FE_Cl_ was also increased significantly, although plasma Cl^−^ concentration was unchanged (Figure 4A). In concordance with increases of FE_Ca_ and FE_Cl_, *Cldn4^−/−^* mice with free access to water and food tended to show increased daily urine volume (p = 0.172) with reduced osmolality (p = 0.017)(Figure 4B). However, 24-hour dehydration caused a sharp decrease of urine volume with increased osmolality in *Cldn4^−/−^* mice to the extent comparable with WT littermates (Figure 4B). Thus, although the basal activity of urine concentration tended to be reduced reflecting the mild impairment of Ca^2+^ and Cl^−^ reabsorption, *Cldn4^−/−^* mice showed no evidence of diabetes insipidus.

### Diffuse Urothelial Hyperplasia Underlies Hydronephrosis in *Cldn4^−/−^* Mice

**Figure 5:**
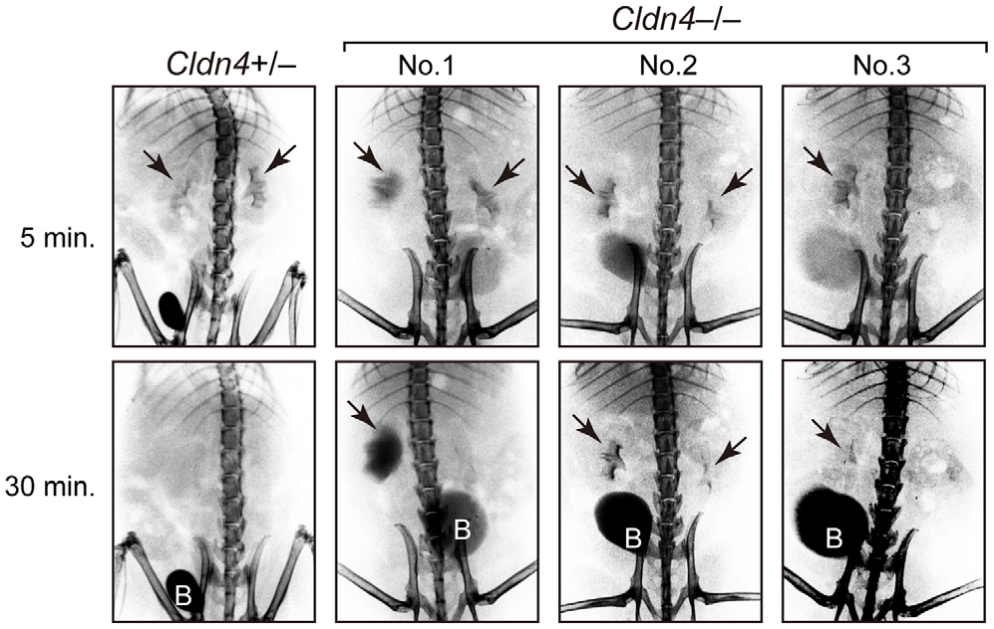
Urinary tract obstruction in *Cldn4^−/−^* mice. Intravenous pyelography (IVP) was performed at 5 and 30 minutes after the injection of Omnipaque in *Cldn4^+/−^* and *Cldn4^−/−^* mice (7–8 months old). Typical examples of *Cldn4^−/−^* mice are indicated: No. 1, 8 months old; No. 2, 7 months old; and No 3., 7 months old. Arrows indicate the depicted pelvises and “B” indicates bladders.

**Figure 6:**
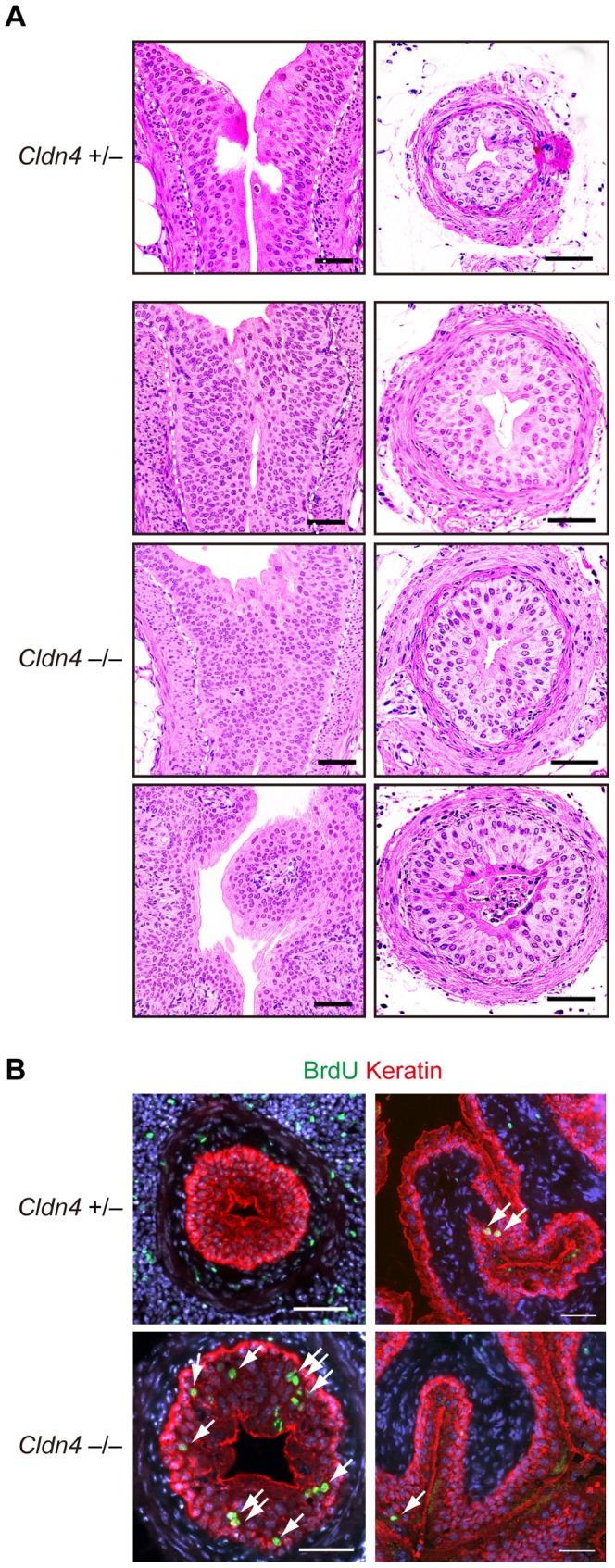
Urothelial hyperplasia with increased BrdU incorporation in *Cldn4* ^−/−^ mice. (*A*) Sections of the uretero-pelvic junction regions (left panels) and lower part of the ureters (right panels) of *Cldn4* ^+/−^ and *Cldn4* ^−/−^ littermate mice (8–19 month old) with no evidence of hydronephrosis were stained with hematoxylin and eosin. Dotted lines indicate the urothelial basal layers. Note the increased urothelial cell numbers and layers. Bars, 50 µm. (*B*) *Cldn4* ^+/−^ and *Cldn4* ^−/−^ littermate mice (12 months old) were injected with BrdU (1 mg/animal) intraperitoneally for 4 consecutive days, and the sections of the ureters (left) and bladders (right) were three-color immunostained with anti-BrdU (green, arrows), anti-keratin (red), and DAPI (blue). Bars, 50 µm.

We then investigated the possibility of postrenal causes for the development of hydronephrosis. IVP analysis revealed that the majority of *Cldn4* ^−/−^ mice at 5–8 months of age exhibited delayed excretion of Omnipaque compared with *Cldn4* ^+/−^ mice at variable levels, including a marked retention at unilateral pelvis (Figure 5, No. 1), delayed clearance at both sides (Figure 5, No. 2), and complete failure of unilateral pelvic depiction (Figure 5, No. 3). The results suggested urinary tract obstruction in *Cldn4* ^−/−^ mice prior to hydronephrosis and prompted us to carefully examine the histology of the urothelium. It was found that the urothelial layers of the renal pelvic region and ureters were thickened diffusely with markedly increased cellularity and layers in *Cldn4* ^−/−^ mice prior to the development of hydronephrosis (Figure 6A). Although papillomatous protrusions were observed occasionally in the pelvis, no overt tumor formation was encountered (Figure 6A). There was no evidence of inflammatory reactions either, such as inflammatory cell infiltration and fibrosis. To directly investigate the proliferation rates of urothelial cells *in situ*, we examined BrdU uptake in the urothelium after BrdU injection for 4 consecutive days. Although BrdU staining was rarely observed in the urothelial cells in *Cldn4* ^+/−^ mice, both the pelvic and ureteral urothelial cells of *Cldn4* ^−/−^ mice with no overt hydronephrosis showed significant incorporation of BrdU (Figure 6B). The bladder urothelial cells of the same *Cldn4* ^−/−^ mice, however, showed no evidence of hyperplasia and rarely incorporated BrdU similar to *Cldn4* ^+/−^ mice (Figure 6B). The results suggest that the pelvic and ureteral urothelial cells in *Cldn4* ^−/−^ mice show increased proliferation as they age, leading to the urothelial hyperplasia and progression of upper urinary tract obstruction.

## Discussion

Nephropathy due to progressive hydronephrosis can be caused by various renal as well as postrenal causes, and a number of genetically engineered mouse models have been described. ClC-K1 and AQP-2, which mediate the transepithelial transport of chloride and water, respectively, in the renal nephrons, play a crucial role in urine concentration, and the ablation of *Clck1* or *Aqp2* or mutation of *Aqp2* results in the rapid development of nephrogenic diabetes insipidus followed by hydronephrosis [31], [32], [33], [34]. On the other hand, ablation of uroplakin genes (*UPII, UPIIIa*) responsible for the formation of UPs covering the surface of the umbrella cells of the urinary tract causes increased urothelial leakiness and hyperplasia, leading to obstructive hydronephrosis [35], [36]. In the current study, we developed *Cldn4* ^−/−^ mice and found that *Cldn4* deficiency also resulted in the development of hydronephrosis. The hydronephrosis developed unilaterally with no overt clinical signs, but the disease progressed bilaterally in a significant proportion of *Cldn4* ^−/−^ mice with age, causing increased mortality.

A recent report indicates that Cld4 is physically associated with Cld8 at TJs in a cultured collecting duct cell line [14]. In *Cldn4* ^−/−^ mice, Cld8 expression at TJs was greatly reduced specifically in collecting duct epithelial cells, in agreement with the coordinated expression of Cld4 and Cld8 in the normal nephron *in vivo*. In any case, the TJ structures were apparently preserved in both collecting duct and urpthelium of *Cldn4* ^−/−^ mice, as judged by the unaffected localization of TJ-associated ZO-1. We found that the *Cldn4* ^−/−^ tubular epithelial cells and urothelium showed markedly enhanced expression of Cld3 and Cld7 at TJs, respectively, which were detected diffusely and only marginally in *Cldn4* ^+/−^ mice. Such a compensatory or back-up localization of other Cld members at TJs has not been reported in other Cld-deficient mice. The overall protein levels of Cld3, Cld7 and Cld8 were unchanged in the kidneys and urothelium of *Cldn4* ^−/−^ compared with *Cldn4* ^+/−^ mice, and thus it was strongly suggested that the effects were due to the altered localization of other preexisting claudin members rather than induction or repression of the expression. Claudins are not always confined to the TJs, but may be found significantly at other subcellular locations such as basolareral membrane and intracellular domains [3]. Although the function of such claudins outside TJs is not well understood, our current results suggest that different Cld members have distinct priorities for accumulating at TJs in specific tissue epithelia and claudins outside TJs may localize at TJs in compensation for the original Clds in certain conditions. The compensatory effects might be influenced by the preference of physical interaction among given Cld members in each tissue.

Cld4 is expressed at the selective segments of nephrons, including the thin descending and ascending loops of Henle, connecting tubule, and collecting duct as well as the entire urothelium from the pelvis to the bladder [37](current results). Accumulating evidence indicates that Clds at TJs of the renal nephrons contribute to the transport of selected ions via a paracellular route [13], [38], [39]. Thus, Cld2 at the proximal tubule promotes the reabsorption of cations (Na^+^, Ca^2+^) by enhancing the paracellular leakiness [13], [38], whereas Cld4 has been shown to act as a paracellular Cl^−^ channel in the collecting duct cell lines such as M-1 and mIMCD3 cells [14]. Current results indicated that *Cldn4* ^−/−^ mice showed selective increase of FE_Cl_, in agreement with the proposed role of Cld4 in the paracellular reabsorption of Cl^−^ in the collecting duct. The reason why FE_Ca_ was also increased in *Cldn4* ^−/−^ mice remained to be investigated. One possibility is that compensatory expression of Cld3, which is shown to be a sealing component of TJs for ions and either charged and uncharged solutes [40], may inhibit Ca^2+^ reabsorption. Secondary effects of decreased Cl^−^ reabsorption may also be considered. In either case, *Cldn4* ^−/−^ mice with free access to water and food tended to show increased urine volume with diminished osmolality, probably reflecting the increase of FE_Ca_ and FE_Cl_. However, *Cldn4* ^−/−^ mice had completely normal capacity for concentrating urine upon dehydration with no evidence of nephrogenic diabetes insipidus, and thus it was quite unlikely that the progressive hydronephrosis in *Cldn4* ^−/−^ mice was nephrogenic.

IVP examination indicated that the urinary flow was retarded to variable extents in *Cldn4* ^−/−^ mice prior to the overt development of hydronephrosis. The effect was observed unilaterally in many cases, suggesting the obstructive changes in the upper urinary tract. Cld4 is expressed along the entire urinary tract with many other Clds [23], [30], [41] and is localized at the TJs of the uppermost umbrella cells as well as throughout the plasma membranes of the underlying urothelial cells. In the urothelium of *Cldn4* ^−/−^ mice, Cld7 expression was specifically enhanced at TJs, while the expression profiles of Cld3 and other Clds were unchanged. The effect was again attributable to the accumulation of preexisting Cld7 at TJs rather than the upregulation of Cld7 protein expression, and it was indicated that the types of compensatory Clds for Cld4 at TJs depended on the context of epithelial cell types. Probably due to the compensatory effect, the TJ formation in the urothelium was apparently unaffected in *Cldn4* ^−/−^ mice, with grossly normal barrier function. However, it was found that the urothelium of the renal pelvis and ureters showed diffuse hyperplasia and thickening in *Cldn4* ^−/−^ mice as they aged. The BrdU labeling experiments *in vivo* indicated that the urothelial cells in *Cldn4* ^−/−^ mice showed markedly increased BrdU uptake with 4-day pulsing, whereas BrdU^+^ cells were rarely detected in the *Cldn4* ^+/−^ urothelium. The effect was apparently “sterile,” and no associated inflammation was observed. The finding was quite surprising, considering the slow turnover rate of urothelial cells (∼3–6 months) in normal mice [23]. The change was observed prior to the development of hydronephrosis and thus was unlikely due to a secondary effect.

The mechanism for the deregulated urothelial cell proliferation in *Cldn4* deficiency remains to be investigated. Of note, *Cldn15* ^−/−^ mice develop mega-intestine with nonneoplastic, deregulated increase of intestinal epithelial cells [18], [42]. The effects may be indirect ones caused by the altered cell microenvironment, such as ion concentrations, due to the changes of paracellular leakiness [43]. Alternatively, Clds may directly regulate cell proliferation. It was reported that Cld2 expressed in colon cancer cells promoted EGFR-mediated proliferation *in vitro* [20], [21], and we reported that Cld4 in the thymocytes was capable of enhancing the TCR-mediated ERK activation [22]. In this regard, it is noted that the defect of uroplakins, which form UP crystals covering the apical surface of umbrella cells, also results in the urothelial hyperplasia of ureters, leading to rapid progression of hydronephrosis [35], [36]. Because the urothelium of *Cldn4* ^−/−^ mice showed normal UPs, the effect should be independent of the UPs. While UPs cover the apical surface of the uppermost umbrella cell layer, Cld4 is localized not only at the TJs between the umbrella cells but distributed diffusely throughout plasma membranes of underlying urothelial cell layers. The apical surface of umbrella cells is associated with heparin-binding epidermal growth factor (EGF), and the smooth muscle cells surrounding the urothelium also produce EGF [44], [45]. Thus, it may be possible that UPs and Cld4 regulate the availability of EGF and/or the signaling of EGFR. In either case, these results suggest that Cld4 and UPs independently regulate homeostatic urothelial cell proliferation. Relatively late onset and chronic progression of hydronephrosis in *Cldn4* ^−/−^ mice, as opposed to early and rapid progression in *UP* ^−/−^ mice, may be due in part to the compensatory function of Cld7 at TJs. Although we have not observed overt tumor development in *Cldn4* ^−/−^ mice, effects of urinary chemical carcinogens are under investigation.

In conclusion, we have demonstrated that *Cldn4* ^−/−^ mice develop chronic urothelial hyperplasia and eventually overt hydronephrosis. Our results indicate that Clds play an important role in maintaining urothelial integrity and provide insights into the function of Clds in tissue homeostasis.

## Materials and Methods

### *Cldn4* ^−/−^ Mice

A targeting vector (Figure S1A) was introduced into D3a2 embryonic stem (ES) cells [46]. Correctly targeted ES cells (Figure S1B) were injected into C57BL/6 blastocysts and transferred into foster mothers to develop heterozygous mice. These mice were further mated with CAG-cre transgenic mice [47] to delete the neomycin cassette and floxed *Cldn4*. Mice were maintained in specific pathogen-free conditions at Kyoto University’s Laboratory Animal Center in accordance with University guidelines.

### Antibodies

The following antibodies were used: anti-Cld2, anti-Cld4, Alexa Fluor 488–conjugated anti-Cld4, anti-Cld13 (Invitrogen), anti-Cld3 (Novus), anti-Cld7, anti-TRPV5 (Abcam), anti-Cld8, anti-Cld12 (IBL), anti-AQP1 (Millipore), anti-ClC-K (Alomone Labs), anti-THP (Biomedical Technologies), anti-AQP2 (a kind gift from Dr. Y. Noda, Tokyo Medical and Dental University, Tokyo), anti-pan-Keratin (DAKO), anti-E-Cadherin (Takara), anti-β-actin (Santa Cruz Biotechnology), Alexa Fluor 488–conjugated anti-BrdU (BD Bioscience) and anti- ZO-1 (obtained from the Developmental Studies Hybridoma Bank, The University of Iowa, USA). Secondary reagents were as follows: Alexa Fluor 488–conjugated streptavidin, donkey anti-rabbit IgG (Invitrogen), Cy3-conjugated donkey anti-rat IgG and anti-rabbit IgG (Jackson), Horse-radish peroxidase-conjugated anti-rabbit IgG (GE healthcare) and anti-goat IgG (KPL).

### Immunostaining

Tissue immunostaining was performed as described before [48]. Tissues were snap-frozen with liquid nitrogen in OCT compound (Sakura), and tissue sections (6-µm thickness) were fixed with 95% ethanol followed by 100% acetone. The sections were blocked and incubated with primary antibodies, followed by secondary antibodies or reagents. For BrdU staining, mice were injected intraperitoneally with BrdU (1.0 mg/animal) for 4 consecutive days, and the tissue sections were first stained with pan-keratin followed by fixation in 4% paraformaldehyde/phosphate-buffered saline (PBS). After washing, sections were treated with 4 N HCl for 20 minutes, neutralized with 0.2 M sodium borate (pH 8.5), and incubated with Alexa Fluor 488–conjugated anti-BrdU monoclonal antibody. The samples were examined with a microscope (Carl Zeiss), and the images were processed with Photoshop.

### *In vivo* Permeability Assay

Anesthetized mice were injected with sulfo-NHS-SS-biotin (606.9 Da, 1 mg/PBS containing 1 mM CaCl_2_ and methylene blue) into the bladder using a 27-gauge needle. After 20 minutes, mice were sacrificed, and the tissues were subjected to immunostaining. Biotin was detected with Alexa Fluor 488–conjugated streptavidin.

### Intravenous Pyelography

Mice were anesthetized and injected intravenously with Omnipaque (Daiichi Sankyo, 3 µl/g of body weight), followed by serial radiographs at 5, 15, and 30 minutes with the use of SOFTEX CMB-2(SOFTEX).

### Electron and Scanning Microscopy

For transmission electron microscopy, the tissues were fixed with 4% paraformaldehyde and 2% glutaraldehyde in PBS, post-fixed with osmium tetroxide, embedded in Epon, and examined under a HITACHI H-7650 microscope. For scanning electron microscopy, tissues were fixed as for transmission electron microscopy, critical-point dried, and examined with a HITACHI H-4700 microscope.

### Plasma and Urine Analysis

Mice were kept in metabolic cages for 24 hours with free access to water and food. Urine samples were collected twice at an interval of 1 week and the average values were used for the statistical analysis. After the second urine collection, mice were sacrificed and whole blood was collected. Urine and serum ions were measured with an autoanalyzer (Hitachi-7180, Hitachi Instruments), and urine osmolality was measured by freezing-point depression osmometry (OM-6030, OM-6050, and OM-6060, Arkray). For the urine concentration test, urine samples were collected after animals were deprived of water for 24 hours.

### Histology

Tissues were fixed in 10% formalin neutral buffer solution (Wako) and embedded in paraffin, and the sections were stained with hematoxylin and eosin.

### Immunoblotting

Kidneys were homogenized with FastPrep24 instrument (MP-biomedicals) in RIPA buffer containing protease inhibitors. The mucosal layer of bladders was mechanically removed from muscle layer and lysed in lysing buffer (20 mM HEPES buffer pH 7.5, 150 mM NaCl, 1% Triton X-100, 1 mM EGTA, 1 mM EDTA, 1.5 mM MgCl2, 10% glycerol, 10 mM sodium pyrophosphate) containing protease inhibitors. Immunoblotting was performed as described before [49].

### Quantitative RT-PCR

Kidney and bladder mucosa were frozen in liquid nitrogen and homogenized with FastPrep24 in TRIzol reagent (Invitrogen). RNA was extracted and transcribed into cDNA with Super-Script III (Invitrogen). Real-time PCR was performed with a LightCycler SYBR Green I marker kit on a LightCycler instrument (Roche). The transcripts of each gene were normalized to those of cyclophilin. Primer sequences for *Cldns* were described before. [22].

## Supporting Information

Figure S1 **Establishment of** ***Cldn4*** **^−/−^ mice.** (*A*) Schematic representation of the targeting vector. E, *Eco*RI digestion site. (*B*) A targeted allele was confirmed by Southern blotting analysis of *Eco*RI-digested genomic DNA using an indicated DNA probe. (*C*) Primer sets for detecting WT and deleted alleles of *Cldn4*.
(TIF)

Figure S2 **Expression profiles of Cld4 in normal mouse renal nephrons and urothelium.** (*A*) Sections of the kidneys of normal B6 mice were two-color immunostained with anti-Cld4 (green) and established markers for various segments of nephrons (red), including AQP1 (proximal tubule and thin descending loop of Henle), CIC-K (thin ascending loop of Henle), THP (thick ascending loop of Henle), AQP2 (connecting tubule and collecting duct), and TRVP5 (connecting tubule). Arrows, Vasa recta. Bars; 20 µm. (*B*) Schematic Cld4 expression profile in nephrons and urothelium is illustrated based on the results.
(TIF)

Figure S3 **Protein and mRNA expression of other Cld members in** ***Cldn4*** **^−/−^ kidneys.** (*A*) *Cldn4* ^+/−^ and ^−/−^ kidneys were lysed and immunoblotted with the indicated antibodies. (*B*) RNA was extracted from *Cldn4* ^+/−^ and *Cldn4* ^−/−^ kidneys and relative *Cldns* transcripts were assessed by qPCR The means of triplicate analysis are shown. The values are mean±SEM. 3-months old mice were used. L and R indicate left and right kidney respectively.
(TIF)

Figure S4 **Protein expression of other Cld members in** ***Cldn4*** **^−/−^ urothelium.** *Cldn4* ^+/−^ and *Cldn4* ^−/−^ bladder mucosa were lysed and immunoblotted with the indicated antibodies. 3-months old mice were used.
(TIF)
