# Supplementary figures and images for: Claudin-4 Deficiency Results in Urothelial Hyperplasia and Lethal Hydronephrosis
Source: PLoS One. 2012 Dec 21;7(12):e52272. doi: 10.1371/journal.pone.0052272 (PMC3528782; doi:10.1371/journal.pone.0052272)

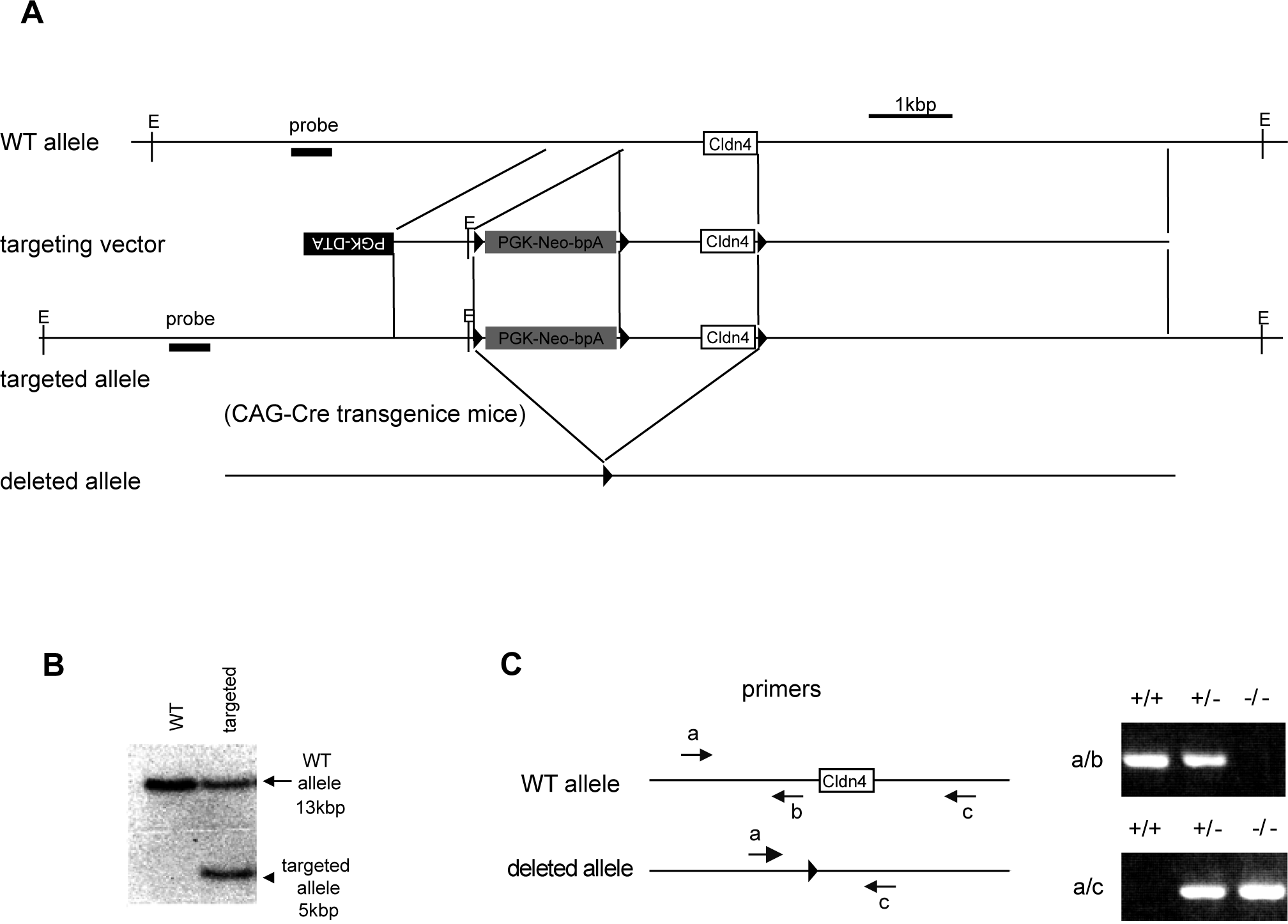

Supplement: Figure S1 — Establishment of Cldn4 −/− mice. (A) Schematic representation of the targeting vector. E, EcoRI digestion site. (B) A targeted allele was confirmed by Southern blotting analysis of EcoRI-digested genomic DNA using an indicated DNA probe. (C) Primer sets for detecting WT and deleted alleles of Cldn4. (TIF) [file pone.0052272.s001.tif]

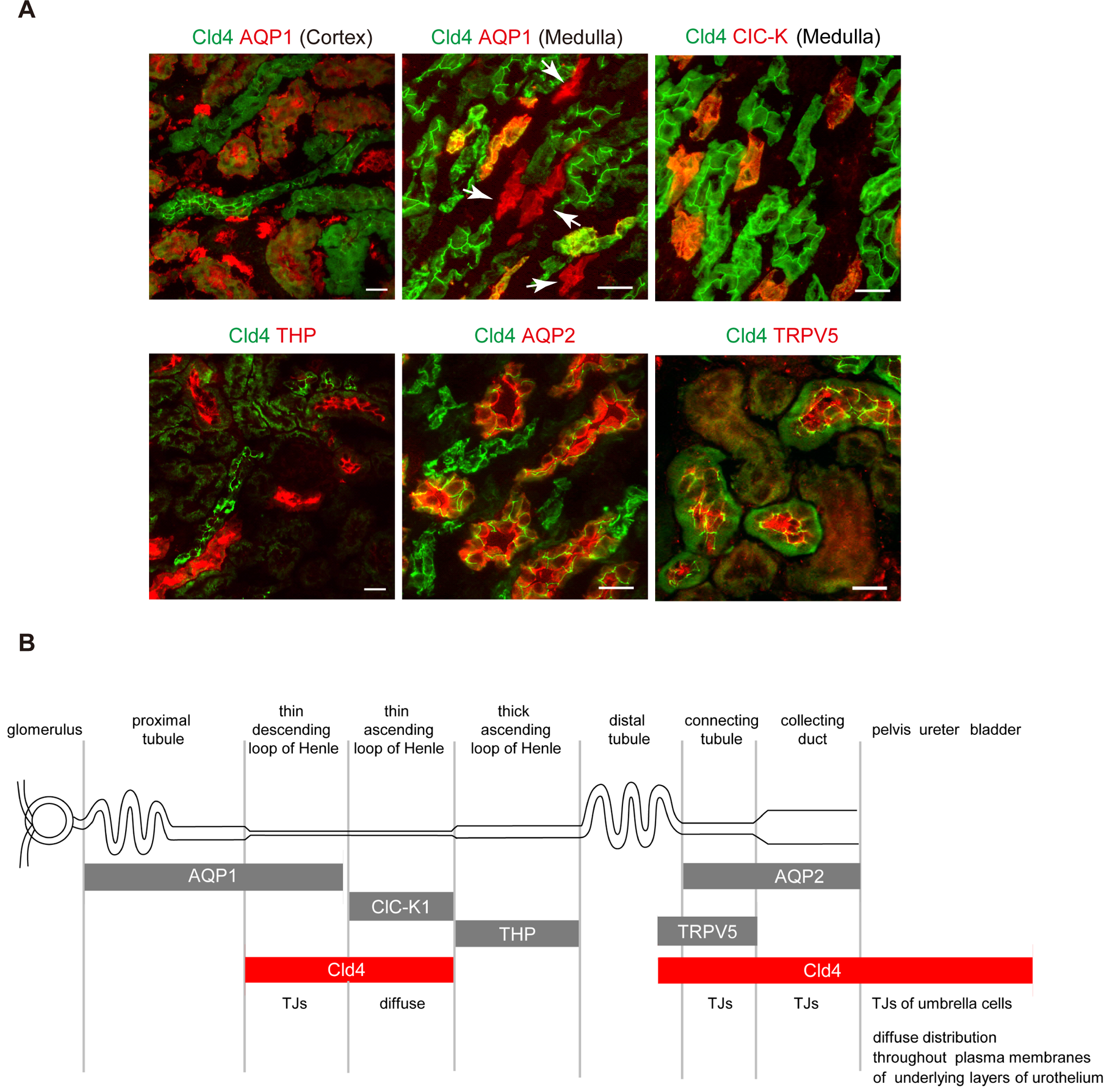

Supplement: Figure S2 — Expression profiles of Cld4 in normal mouse renal nephrons and urothelium. (A) Sections of the kidneys of normal B6 mice were two-color immunostained with anti-Cld4 (green) and established markers for various segments of nephrons (red), including AQP1 (proximal tubule and thin descending loop of Henle), CIC-K (thin ascending loop of Henle), THP (thick ascending loop of Henle), AQP2 (connecting tubule and collecting duct), and TRVP5 (connecting tubule). Arrows, Vasa recta. Bars; 20 µm. (B) Schematic Cld4 expression profile in nephrons and urothelium is illustrated based on the results. (TIF) [file pone.0052272.s002.tif]

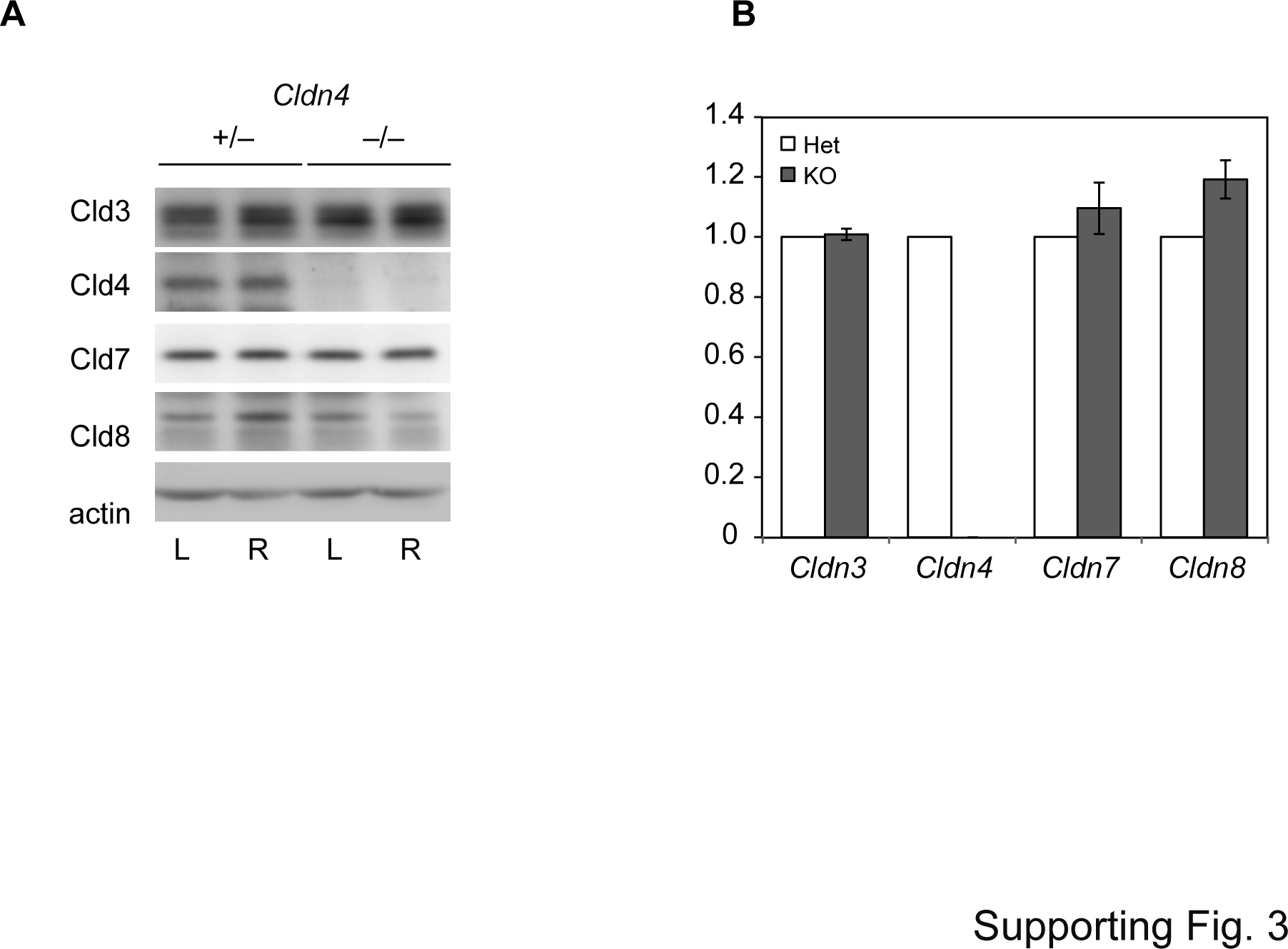

Supplement: Figure S3 — Protein and mRNA expression of other Cld members in Cldn4 −/− kidneys. (A) Cldn4 +/− and −/− kidneys were lysed and immunoblotted with the indicated antibodies. (B) RNA was extracted from Cldn4 +/− and Cldn4 −/− kidneys and relative Cldns transcripts were assessed by qPCR The means of triplicate analysis are shown. The values are mean±SEM. 3-months old mice were used. L and R indicate left and right kidney respectively. (TIF) [file pone.0052272.s003.tif]

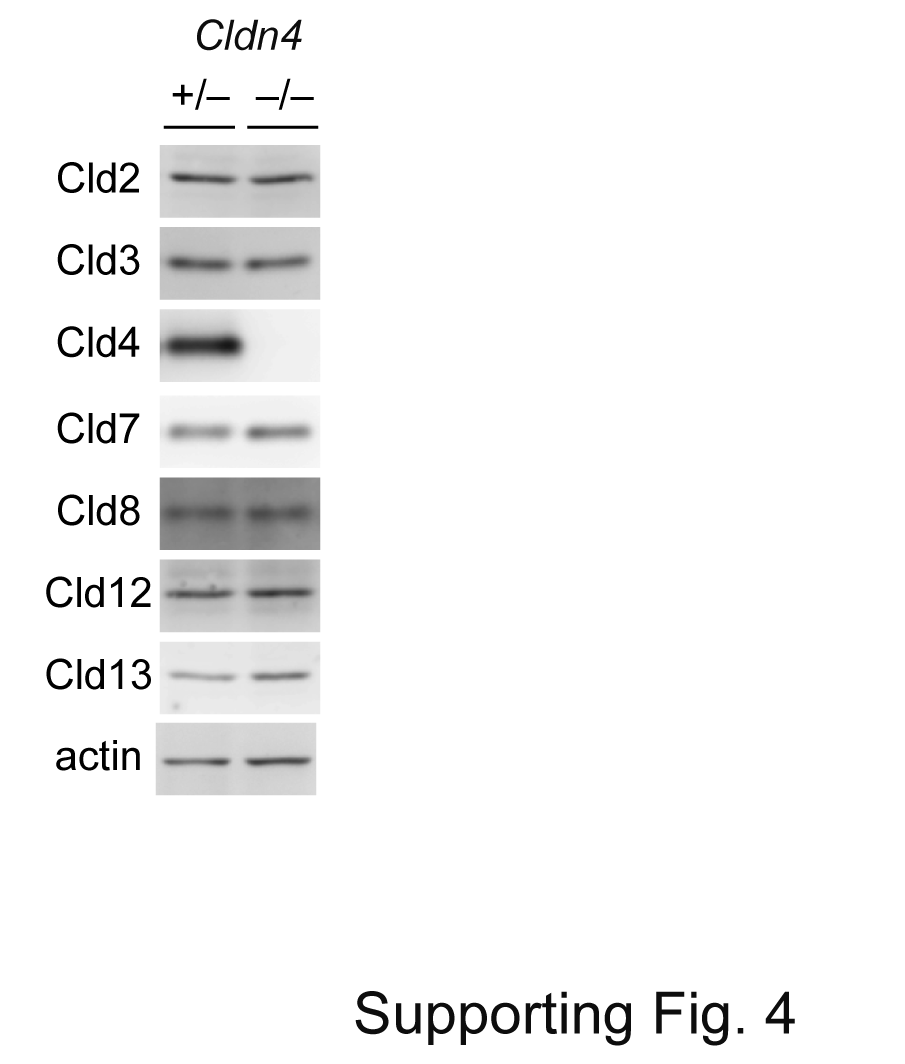

Supplement: Figure S4 — Protein expression of other Cld members in Cldn4 −/− urothelium. Cldn4 +/− and Cldn4 −/− bladder mucosa were lysed and immunoblotted with the indicated antibodies. 3-months old mice were used. (TIF) [file pone.0052272.s004.tif]
